# Timing of cerebral damage in molybdenum cofactor deficiency: A meta-analysis of case reports

**DOI:** 10.1016/j.gimo.2024.101853

**Published:** 2024-05-24

**Authors:** Elise A. Ferreira, Floris C. Hofstede, Hanneke A. Haijes-Siepel, Klaske D. Lichtenbelt, Lou Pistorius, Monique G.M. de Sain-van der Velden, Peter G.J. Nikkels, Maarten H. Lequin, Linda S. de Vries, Saskia N. van der Crabben, Peter M. van Hasselt

**Affiliations:** 1 Department of Pediatrics, Emma Children's Hospital, Amsterdam UMC, University of Amsterdam, Amsterdam, The Netherlands; 2 United for Metabolic Diseases (UMD), The Netherlands; 3 Department of Metabolic Diseases, Wilhelmina Children’s Hospital, University Medical Center Utrecht, Utrecht, The Netherlands; 4 Department of Genetics, University Medical Center Utrecht, Utrecht, The Netherlands; 5 Department of Gynecology, University Medical Center Utrecht, Utrecht, The Netherlands, currently Mediclinic Panorama, Cape Town, South Africa; 6 Section Metabolic Diagnostics, Department of Genetics, University Medical Center Utrecht, Utrecht, The Netherlands; 7 Department of Pathology, University Medical Center Utrecht, Utrecht, The Netherlands; 8 Department of Radiology, University Medical Center Utrecht, Utrecht, The Netherlands; 9 Department of Neonatology, University Medical Center Utrecht, Utrecht, The Netherlands; 10 Department of Clinical Genetics, Amsterdam University Medical Center, Amsterdam, The Netherlands

**Keywords:** Epileptic encephalopathy, Molybdenum cofactor deficiency, Natural evolution study, Sulfite oxidase deficiency, Meta-analysis of case reports

## Abstract

**Purpose:**

Molybdenum cofactor deficiency (MoCD) classically presents shortly after birth, with neurological symptoms ascribed to postnatal toxicity of accumulating sulphite. Case reports suggest that cerebral damage associated with MoCD may have a prenatal onset.

**Methods:**

A meta-analysis of case reports was performed on individuals with genetically proven MoCD retrieved through a systematic review and in-house search. Cases were categorized as classical or late-onset, based on the time of onset of symptoms. Available cerebral images were scored for the presence of restricted diffusion, pathological signal, subcortical cysts, and atrophy. Estimated onset of each event and the minimal number of events needed to explain the observed imaging abnormalities were deduced by combining age at imaging, type of imaging abnormality, and known natural evolution of the imaging abnormalities.

**Results:**

Of a total of 30 retrieved cases, 21 were classical. Prenatal origin of damage was possible in all classical cases and certain in 11 of 21 (52%). Multiple events were deduced in 5/21 classical cases based on imaging data alone and in 11 of 21 cases when presuming that a postnatal onset of symptoms signifies a recent event. Multiple, but postnatal, events were also described in 3 of 9 late-onset cases.

**Conclusion:**

Prenatal onset of cerebral damage in patients with classical MoCD is more frequently encountered than anticipated. It may have been overlooked by the overwhelming postnatal symptoms erroneously pointing to a single culprit. This insight is important when counseling for prognosis, particularly in the context of considering the timing and anticipated prospects of therapeutic intervention.

## Introduction

Molybdenum cofactor deficiency (MoCD) is a rare autosomal recessive condition of sulfur-containing amino acid metabolism.^1^ It is caused by pathogenic variants in *MOCS1*, *MOCS2*, *MOCS3*, and *GPHN*, respectively (OMIM #252150/#252160/#60927/#615501).^2^ Classically, patients present with signs of irritability, hyper- or hypotonia, intractable seizures, encephalopathy, hyperreflexia, apnea, and feeding difficulties within the first days after birth.^3^

It has been suggested that clinical symptoms result from a rapid postnatal accumulation of sulfite and S-sulfocysteine in blood, urine, and cerebrospinal fluid after the loss of protection through placental filtration.^4^ Sulfite has been shown to deplete intracellular adenosine triphosphate in cultured neuronal cell lines and compromise mitochondrial respiration, whereas S-sulfocysteine (the product of sulfite and cystine), activates N-methyl-D-aspartate receptors.^5^^,^^6^ These mechanisms are thought to be responsible for the specific combination of energy failure and excitotoxic type of cerebral brain damage associated with MoCD.^1^ Not surprisingly, imaging abnormalities in MoCD closely resemble those found in hypoxic ischemic encephalopathy (HIE).^7^, ^8^, ^9^ HIE results from a perinatally impaired cerebral blood flow and oxygen transfer to the cerebrum and is frequently encountered in clinic.^8^^,^^10^ HIE patients show a remarkable resemblance to patients diagnosed with MoCD; therefore, MoCD cases are initially often wrongfully diagnosed as HIE.^8^

The hypothesis that the damaging effects of MoCD only become apparent after birth has recently been questioned by a number of case reports describing characteristic imaging abnormalities associated with MoCD on prenatal ultrasound (US). These observations indicate that maternal clearance of toxic compounds in utero may not always be sufficient to prevent cerebral damage.^1^^,^^6^^,^^11^^,^^12^

The timing of damage is clinically relevant because a treatment option has become available that appears to prevent further cerebral damage.^1^ Assessing the extent to which prenatal damage plays a role is, at first glance, precluded by the fact that cerebral imaging is generally only performed after birth. We therefore wondered whether the natural evolution of radiological features can be used to estimate the time of onset of cerebral damage in patients with MoCD, allowing us to evaluate the presence of prenatal damage, even in imaging performed after birth. Cerebral imaging abnormalities observed in patients with MoCD closely resemble those described after HIE.^3^^,^^13^ Initially extensive bilateral abnormal signal intensity (sometimes sparing thalami and frontal and temporal cortex) is present, suggesting the edema of both gray and white matter. After edema decreases, curvilinear areas of reduced signal intensity appear at the gray/white matter junction. This is the area with the most N-methyl-D-aspartate receptors.^14^ Hereafter, in the final stage, there is a rapid development of extensive (collapsing) cystic leukomalacia together with cortical atrophy, leading to enlarged extra-axial spaces and abnormal signal intensities in the basal ganglia, internal capsule, and centrum semiovale. Each of these radiological features is associated with its own natural evolution. Some may be observed within hours after an event and disappear within days, whereas others take weeks or even months to become visible.

Here, we performed an in-house search expanded with a meta-analysis of imaging data obtained from published case reports to further delineate the disease onset.

## Materials and Methods

### In-house MoCD cases

We retrieved all in-house cases with MoCD diagnosed between January 2008 until March 2023 from our in-house patient database.

In 3 patients, the initial diagnosis was based on clinical suspicion and subsequent postnatal metabolic analyses in plasma and urine. In 2 patient, suspicion of MoCD rose prenatally. In this patient (for the first time) metabolic diagnostics were performed in the amniotic fluid. MoCD was confirmed in all 4 cases through Sanger sequencing. Cases were reviewed and summarized, with an emphasis on imaging data.

Prenatal US, if applicable, was performed using 3-dimensional US and color Doppler (Voluson E8 or E10 system, 2 −6 MHz RM6C transducer) (GE Voluson TM Healthcare). Postnatal cranial US (cUS) was performed using an Aplioi600 or i800 US machine (Canon Medical Systems Europe) with convex 5 to 10 MHz and linear 15 to 18 MHz probes. Magnetic resonance imaging (MRI) was performed on a 1.5T system (Intera or Achieva; Philips Healthcare, Best), and the protocol included sagittal T1-weighted images (slice thickness, 2 mm) and axial T2-weighted images (slice thickness, 2 mm). Between 2010 and 2017, brain MR imaging was performed on a 3T system (Achieva; Philips Healthcare), and the recent protocol included conventional sagittal T1-weighted imaging (slice thickness, 3 mm), axial 3-dimensional T1-weighted imaging (slice thickness, 2 mm), and axial T2- weighted imaging (slice thickness, 2 mm). Additionally, diffusion weighted imaging and susceptibility weighted imaging were used on postnatal imaging.

### Systematic review

A systematic review was performed according to the “Preferred Reporting Items for Systematic Reviews and Meta-Analyses statements.”^15^^,^^16^ Eligible articles were searched using a combination of Medical Subject Headings and free text, from inception of these databases to November 2021. Studies were identified with the following terms: “Molybdenum/deficiency” (Medical Subject Headings terms) and “Molybdenum cofactor deficiency” (all fields). Three researchers (E.A.F., S.N.v.d.C., and F.C.H.) independently conducted the literature search and reviewed the titles, abstracts, and full-text articles to determine if they met the inclusion criteria. Any conflicts were resolved through discussion with another co-author. Snowballing was performed on references of all included studies to identify additional cases. Cases were included if the following criteria were met: (1) were written in English, (2) had genetic confirmation consisting of ([likely] pathogenic variants in *MOCS1* and *MOCS2*), and (3) had availability of cerebral imaging data (US and/or MRI).

#### Extraction of clinical and radiological data

To perform a meta-analysis of case reports, clinical, genetic, and radiological data of each case described in the included papers were extracted. Symptoms and signs (including the presence of MoCD-specific dysmorphic features: distinct facial features and microcephaly) and their age at onset were collected, as were the genetic data. From each patient data from all cerebral images (imaging abnormalities and the age at imaging) were collected.

#### Classification of disease severity

Cases were categorized based on the time of onset of symptoms as a proxy for disease severity. Patients in whom prenatal imaging revealed intracerebral damage and/or that exhibited symptoms before the 8th day of life were categorized as the classical, patients presenting after the age of 50 days until years of life, were categorized as late-onset.

### Classification of imaging abnormalities

Imaging abnormalities of in-house and published cases were scored for the following abnormalities: (1) pathological signal, (2) restricted diffusion, (3) subcortical cysts, and (4) atrophy. The pathological signal was defined as increased echogenicity on cUS and/or increased signal intensity on T2-weighted sequence on MRI or reduced SI on T1-weighted sequence) in gray and/or white matter. Restricted diffusion was assessed on the b-1000 and ADC maps.

#### Estimating the time of onset of cerebral damage using imaging findings

**Figure 1:**
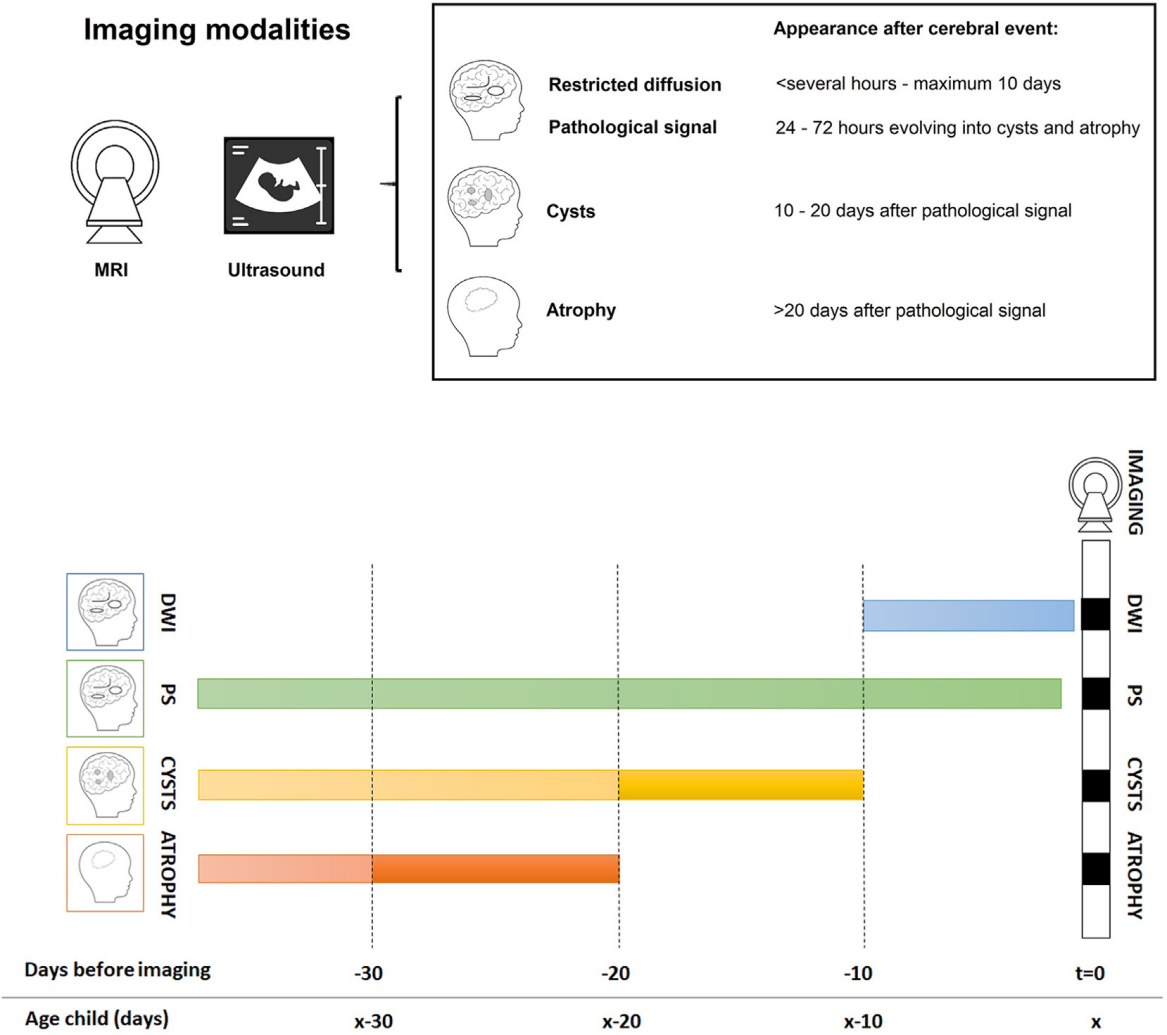
**Timing of onset and resolution of cerebral imaging abnormalities after an event.** Estimation of the moment of onset development cerebral damage associated with molybdenum cofactor deficiency (based on natural evolution of hypoxic ischemic encephalopathy) detected by cerebral ultrasound and/or magnetic resonance imaging (at t = 0). In green (pathological signal, PS); blue (restricted diffusion, DWI); yellow (cysts); and orange (atrophy). PS and atrophy are irreversible types of cerebral damage.

The natural evolution of imaging abnormalities after an acute event was obtained from serial imaging studies in affected HIE neonates and was used to delineate the expected timing of appearance and disappearance of each if the 4 main cerebral imaging abnormality associated with MoCD (Figure 1).^13^^,^^17^

The estimated time of the event was calculated by subtracting the time (days) needed for imaging abnormalities to occur from the age at imaging. A definite prenatal onset was deduced when both the lower and upper onset border of the natural evolution of a type of cerebral damage observed in the patient were of prenatal origin (negative number of days). A definite postnatal onset was deduced from cerebral imaging when both the lower and upper onset border of the natural evolution of all types of cerebral damage observed in the patient of the first imaging result fell within the postnatal range.

#### Minimal number of events needed to explain imaging findings

**Figure 2:**
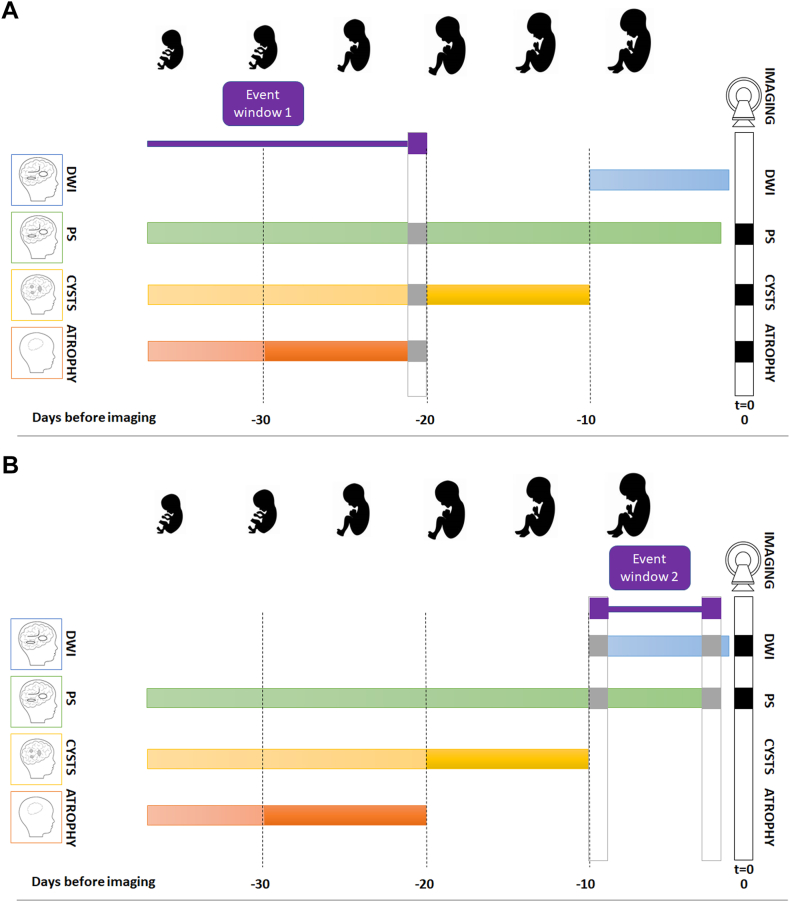
**Estimation of minimal events of disease impact (minimal number of events).** Different cerebral damage types are depicted in blue (DWI), green (pathological signal), yellow (cysts), and orange (atrophy). T = 0 signifies time of imaging (cerebral magnetic resonance imaging or ultrasound). Black boxes indicate the abnormalities found on imaging. A. The presence of cysts and PS together corresponds with an event dating back 20 days or more. B. The presence of DWI and PS together corresponds with a time of event up to 10 days before imaging.

To assess whether or not all radiological findings could be attributed to a single event, we calculated the expected event window of onset of damage for each of the imaging abnormalities. We defined an event window as follows: (age at imaging − minimal time needed to develop imaging abnormalities) − (age at imaging − maximal time for an abnormality to persist). Based on this, we calculated the minimal number of events needed to explain all imaging abnormalities. See Figure 2, for a visualization of this process.

## Results

### In-house cases (case numbers: 1, 9, 10, and 16)

**Figure 3:**
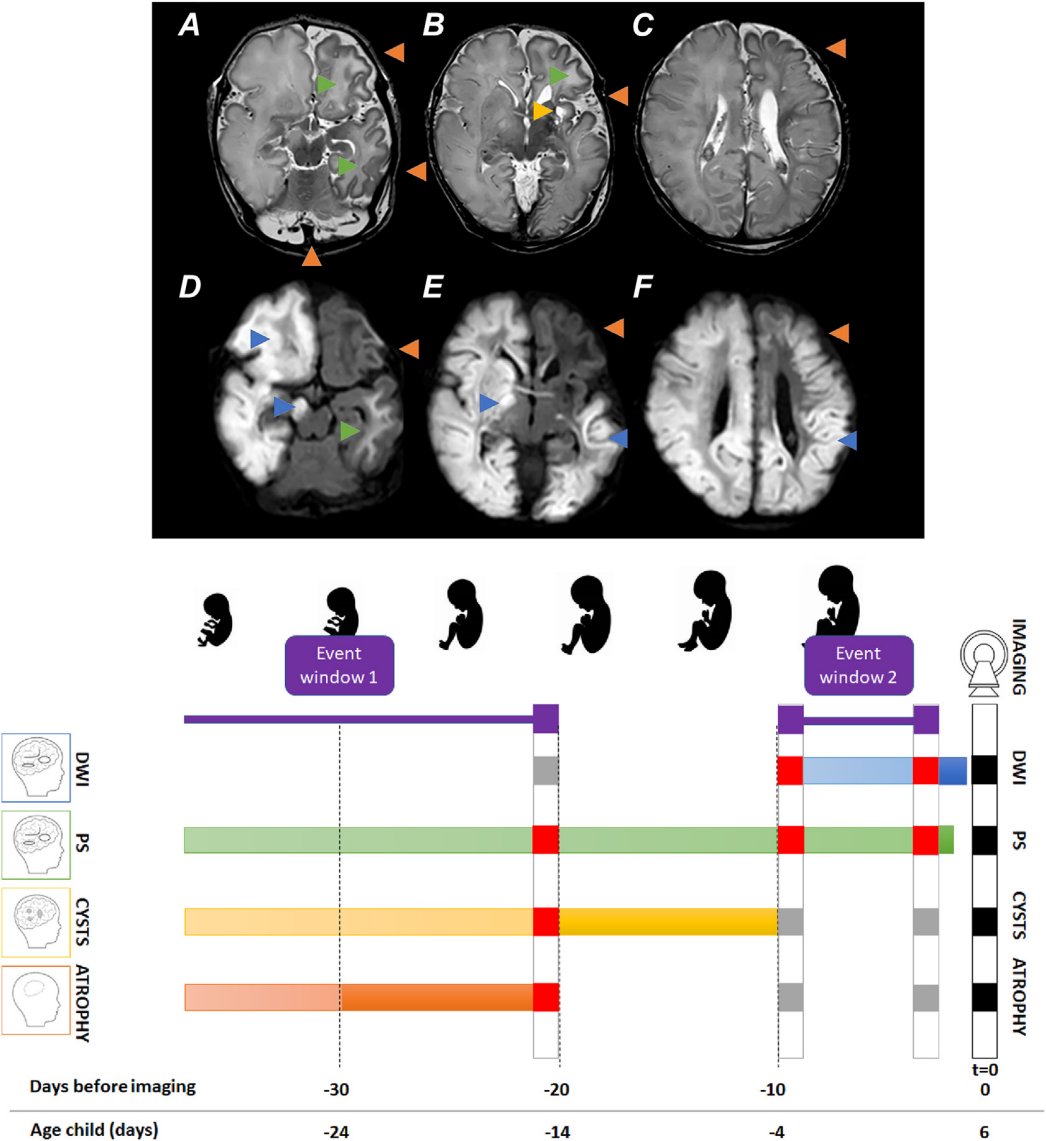
**The estimated onset of cerebral damage of in-house case 16.** Cerebral imaging (magnetic resonance imaging at day 6) of the patient revealing all 4 types of abnormalities. Although the presence of DWI abnormalities can only be explained by a recent event, the presence of atrophy can only be explained by an event dating 20 days or more earlier. The lack of overlap implies that there must have been at least 2 separate events to explain all imaging abnormalities.

Between January 2008 and May 2023, 4 in-house cases were retrieved. See the supplemental file for extended case reports (Supplemental File 1, In-house cases 1, 9, 10 and 16). Three of 4 cases exhibited evidence of antenatal damage. Interestingly, in 1 case, the radiological images could only be reconciled when presuming at least 2 events. Specifically, both swelling (acute toxicity), cystic changes, and atrophy were visible at a single time point (Figure 3). Postmortem analysis, available in 1 (C2, case 9), supported this notion. In this patient, extensive cystic degeneration of the white matter was visible macroscopically. Microscopically, there was severe gliosis, loss of white matter with extensive microglia activation, and the influx of macrophages (as evidenced by positive staining for anti-GFAP, HLA DR, and CD68). These white matter abnormalities were likely to have originated 1 to several weeks before birth. On the other hand, basal ganglia, hippocampi, thalami, and cerebral cortex exhibited extensive cytotoxic edema, along with neuronal loss and some degree of microglial activation. The damage to the basal ganglia, hippocampi, thalami, and cerebral cortex appeared to be of more recent origin, estimated as a few days, around birth.

### Structured literature search and meta-analysis of case reports

**Figure 4:**
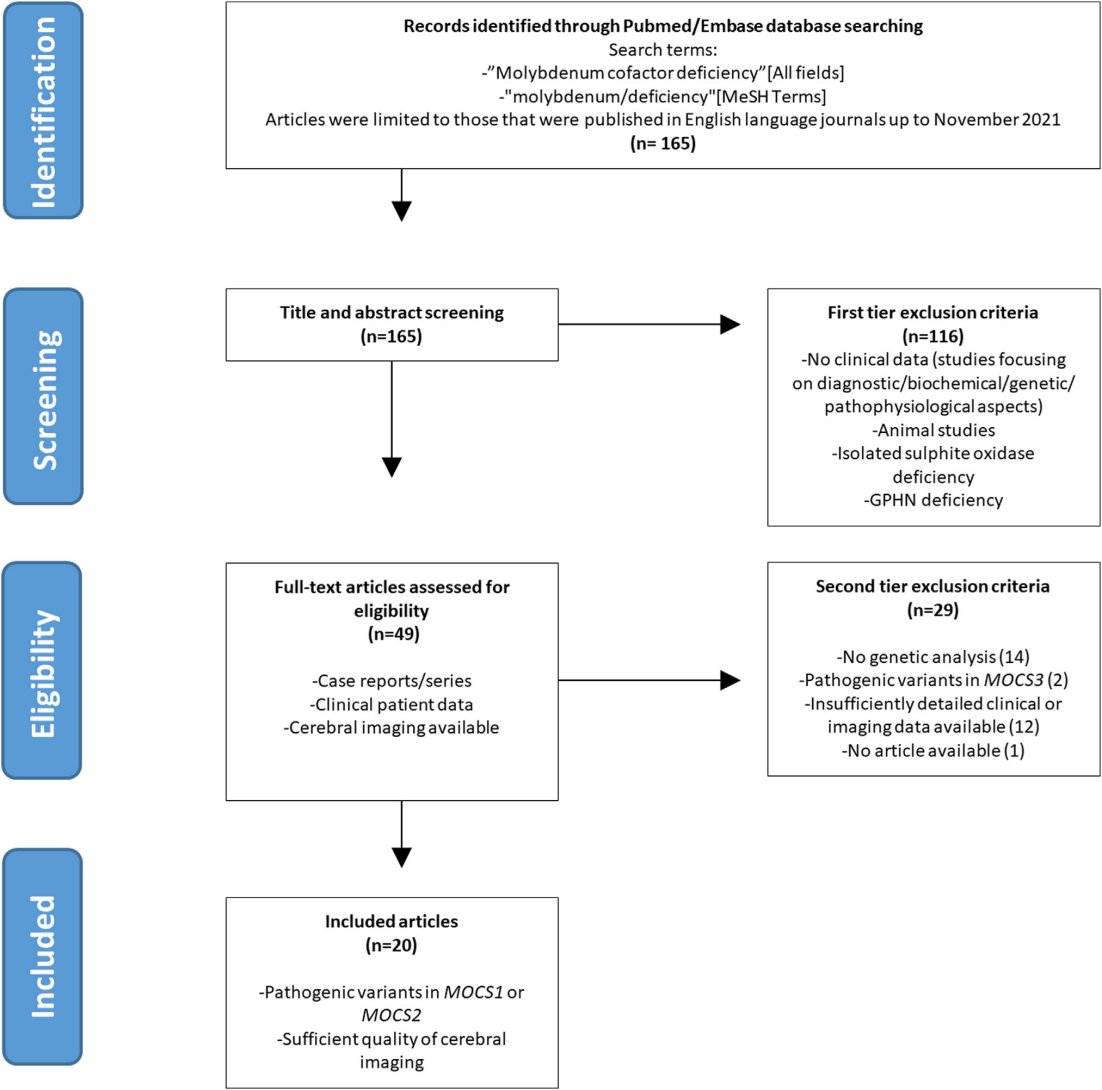
**PRISMA flowchart**.

The initial literature search yielded 165 articles of which 49 articles were retrieved. Of these, 20 articles were included describing 26 cases (Figure 4). These cases, together with the in-house cases were used to perform a meta-analysis of case reports.

### Patient characteristics

Clinical details of all 30 cases are summarized in Table 1, Clinical characteristics. The age at initiation of symptoms ranged from hours from birth to 6 years of age. Twenty-one of the 30 cases had a classical phenotype (cases 1-21; 70%). In 16 of these 21 cases (cases 1-16; 76%) symptoms manifested within the first day of life. Nine of 30 exhibited a late-onset disease course (cases 22-30; 30%) with symptoms only becoming apparent after weeks to years (range 2 weeks-6 years). Posterior fossa abnormalities were observed in 4 of 21 (19%) classical cases and, unexpectedly, in 6 of 9 (67%) late-onset cases. Seven of 21 (33%) classical cases showed dysmorphic features and 2 of 9 (22%) late-onset cases.

**Table 1:** Clinical characteristics

Case (Ref.)	*MOCS1/2*^a^	Specific Variant	Age First Symptoms	Clinical Symptoms	Disease Course (Age)	Dysmorphic Features	Number of Events (Based on Disease Course)
1 (In-house C1)	1	Homozygous NM_001358530.2:c.418+1G>A	1 day	In utero diagnosis, abnormal breathing on day 1	Severe encephalopathy	Full cheeks, deep-set eyes	1
2 (Lubout et al.^11^ 2018)	1	Homozygous c.418+1 G>A; p(?)	1 day	Seizures	Treated with cyclic pyranopterin monophosphate (cPMP): Bayley score 29-35 months, cognitive and motor function P16, head circumference >P98 (41 months)	Macrocephaly	1
3 (Hannah-Shmouni et al.^12^ 2019)	2	Homozygous c.146G>A; p(?)	1 day	Myoclonic jerks	NR		1
4 (Lubout et al.^11^ 2018)	1	Homozygous c.949C>T; p.R317C	1 day	Abnormal EEG, without clinical symptoms	Treated with cPMP: Bayley score 18-26 months, cognitive function P5, motor function P0.5, clumsy walking, head circumference P50 (41 months)		1
5 (Gümüş et al.^18^ 2010)	1	Homozygous c.217C>T; p.R73W	1 day	Feeding difficulties, poor spontaneous activity, seizures	Unexplained cries, irritability, and seizures (3 months); intractable seizures, and developmental delay (4 months)	Triangular face, anteverted nostrils	2 or 3
6 (Carmi-Nawi et al.^19^ 2011)	1	Homozygous c.251-418del; p.(?)	1 day	Seizures (16 hours pp)	Developmental delay, feeding difficulties and failure to thrive (5 months); no eye contact and no social smile, irritability, severe head lag, hypertonia, hyperreflexia (10 months); deterioration of neurological status (11 months); deceased (14 months)		2
7 (Higuchi et al.^20^ 2014)	1	Homozygous c.1643C>A; p.(?)	1 day	Pedaling and crawling seizures	Severe spastic tetraplegia, and epilepsy (2 years)		1
8 (Higuchi et al.^20^ 2014)	1	Homozygous c.1643C>A; p.(?)	1 day	Feeding problems, poor sucking, clonic spasm (29 hours pp)	Severe spastic tetraplegia, and epilepsy (5 years)		1
9 (In-house C2)	1	NM_001358530.2:c.418+1G>A and NM_001358530.2:*c*.*956G*>A(p.Arg319Gln)	1 day	Apnea, cyanosis, jitteriness, seizures, hypoglycemia	Deceased (4 days)		1
10 (In-house C3)	1	NM_001358530.2:c.418+1G>A and NM_001358530.2:c.377G>A(p.Gly126Asp)	1 day	Feeding difficulties, hypertonia, progressive refractory seizures	Deceased (7 days)	Prominent frontal bossing and deep-set eyes	1
11 (Kikuchi et al.^21^ 2012)	2	Homozygous c.265_266delinsCG; p.(∗89Argext∗3)	1 day	Subtle seizures with unusual rowing and pedaling motions, tonic seizures of extremities	Generalized hypertonia, involuntary movements of limbs, hyperreflexia with spasticity in the extremities, arthrogryposis, bedridden, could not sit without support, severe intellectual impairment (12 months)		1
12 (Bayram et al.^22^ 2013)	1	Homozygous AG deletion	1 day	Seizures	Intractable seizures, feeding difficulties (poor suck and swallowing difficulties), severe truncal hypotonia, hyperreflexia (21 days)	Broad nasal bridge, high arched palate and prominent cheeks	2
13 (Sie et al.^23^ 2010)	2	Homozygous c.220C>T; p.(Q74X)	1 day	Myoclonic movements	Respiratory insufficiency (3 days); respiratory, circulatory Insufficiency and deceased (day 11)	Bilateral microphthalmia, wide nasal bridge	1
14 (Per et al.^24^ 2007)	2	Homozygous c.130C>T; p.R44X	1 day	Apnea, bradycardia and hypotonic	Inactive with poor sucking (day 2); poor sucking, unexplained cries, irritability, and seizures (day 50)		2
15 (Veldman et al.^25^ 2010)	1	Homozygous p.G175R (GGG-to-AGG)	1 day	Jerkiness, twitching, and poor sucking	Treated with cPMP: seizures(60 hrs pp); general hypertonia, frequent startle reactions, myoclonic twitching (day 6)		1
16 (In-house C4)	2	NM_001358530.2:c.217C>T(p.Arg73Trp) and NM_001358530.2:c.418+1G>A	1 day	Jitteriness and seizures	Deceased (day 9)		2
17 (Serrano et al.^26^ 2007)	2	Homozygous c.-9-14del23; p(?)	3 days	Feeding difficulties, somnolence, poor spontaneous activity, hypotonia, apneas, and seizures	Lethargic, axial hypotonia, peripheral hypertonia, myoclonic spasms and hyperreflexia (day 5)		1
18 (Vijayakumar et al.^27^ 2011)	2	c.226G>A/c.226G>A	3 days	Encephalopathy and neonatal seizures	NR	Frontal bossing, adducted thumb, macrocephaly	1
19 (Lin et al.^28^ 2021)	2	Homozygous c.168_169del	3 days	Feeding difficulties, strenuous breathing, refractory convulsions, dystonia and areflexia	NR		1
20 (Nagappa et al.^29^ 2015)	2	Homozygous c.252dup; p.(I85Hfs∗2)	5 days	Generalized seizures	Progressive stiffness with intermittent posturing of neck and trunk (6 months); lens dislocation (4 months)		2
21 (Yoganathan et al.^8^ 2018)	2	Homozygous c.218T>C; p.(L73)	8 days	Irritable, inconsolable cry, multifocal clonic seizures and poor spontaneous limb movements	Poor feeding and lethargy (day 8); episodes of myoclonus and seizures (2 months); deceased (9 months)		2
22 (Yoshimura et al.^30^ 2019)	1	Homozygous c.949C>T; p.(R317C)	14 days	Feeding difficulties, irritable, opistothonus	Loss of head control, progressive microcephaly (7 months); convulsive seizures (5 years); tracheobronchomalacia (12 years)	Frontal protrusion, microcephaly, vertical face, long philtrum, thick lips, and long palpebral fissure	3
23 (Yoshimura et al.^30^ 2019)	1	Homozygous c.949C>T; p.(R317C)	14 days	Feeding difficulties, irritable	Opistothonus with extension of limbs, hypertonic, persistent generalized muscle contractions, seizures, psychomotor developmental delay (3 months)		2
24 (Stence et al.^31^ 2013)	2	Homozygous c.564+1G>A	26 days	Infantile spasms, lethargy	Poorly controlled infantile spasms, profound global developmental delay, severe feeding difficulties and loss of gag reflex (5 months)		2
25 (Lee et al.^32^ 2021)	2	Homozygous c.16C > T; p.(Gln6Ter)	“After birth”	Irritable cry, feeding difficulty	Increased tonicity (1 month); myoclonic seizures (8 months); poor eye contact, poor swallowing, generalized hypertonicity and rigidity, brisk deep tendon reflexes, bilaterally positive Babinski signs and ankle clonus, head lag, regression (20 months)	Coarse face with a wrinkled forehead and hair growth, prominent philtrum nasi, and sagging skin and lens subluxation	3
26 (Sass et al.^33^ 2010)	1	Homozygous c.667_668insCGA; p.(Gly223delinsAlaSer)	6 weeks	Screaming episodes, poor feeding and fever, frequent seizures	Irritable, hypertonic, opisthotonus posture, no head control or visual contact (29 months); sucking and swallowing severely compromised, hypertonicity, frequent infections and recurrent seizures (41 months); lens dislocations (44 months)		1
27 (Vijayakumar et al.^27^ 2011)	1	Homozygous c.260C>T; p.(R91W)	8 months	Developmental delay and seizures after intercurrent illness	Lens subluxation, seizures, neuroregression after intercurrent illness (26 months)		2
28 (Lee et al.^32^ 2021)	2	Homozygous c.16C > T; p.G6X	17 months	Upper tract infection, head and trunk waving, left clenched hand and inability to stand or walk	Right-sided hemiparesis, altered level of consciousness, head deviation to the left side and general weakness (17 months and 2 weeks)		2
29 (Vijayakumar et al.^27^ 2011)	1	Homozygous c.1102+6T>C; p.(?)	24 months	Global developmental delay	Lens subluxation (66 months)		1
30 (Alkufri et al.^34^ 2013)	2	c.564G>C/c.726-727delAA	6 years	Normal development	Lens dislocation (6 years); torticollis, parkinsonism and dystonia (23 years)		2

*NR*, not reported; *PP*, postpartum.

a Genetic information is directly copied from related article and has not been validated by our group.

### Disease onset deduced from cerebral images

Cerebral images of all cases were analyzed, and abnormalities were scored (Table 2, Typical cerebral MoCD damage detected by imaging modalities) (see Supplemental File 2, Supplemental information on the onset of damage and multiple hits explained per case, for elaborate explanations on the determined onsets of cerebral damage per patient). The prenatal onset of damage was definite in 11 of 21 classical cases (52%) (cases 1-8, 16-18). In 5 cases, this was based on antenatal imaging (cases 1-4 and 6), in 6 cases prenatal origin was deduced based on the early presence of specific types of postnatal cerebral damage (cases 5,7, and 8 and 16-18). A prenatal onset of cerebral damage could not be excluded based on imaging in any of the 21 classical cases. Conversely, in none of the late-onset cases a prenatal onset was considered definite.

**Table 2:** Typical cerebral MoCD damage detected by imaging abnormalities

Case (Reference)	Age at Cerebral US/MRI/CT	Cerebral or Cerebellar Abnormalities						Prenatal?	Minimal Number of Events (Based on Imaging)
		Pathological Signal	Restricted Diffusion	Periventricular/Subcortical Cysts	Atrophy	Posterior Fossa Abnormalities^a^	Other		
1 (In-house C1)	US at 34 weeks GA			+	+	+		y	1
	MRI at 35 weeks GA			+	+	+			
	US day 1			+	+	+			
	MRI at day 2			+	+	+			
	MRI at 3 months				+	+	s.h.		
2 (Lubout et al.^11^ 2018)	MRI at 20 weeks GA							Y	1
	MRI at 29 weeks GA								
	MRI at 34 weeks GA								
	MRI at 36 weeks GA	+							
	MRI at day 1	+							
	MRI at day 22	+							
	MRI at 47 months				+				
3 (Hannah-Shmouni et al.^12^ 2019)	US at 25 weeks GA					+		Y	1
	MRI at day 1			+	+	+			
4 (Lubout et al.^11^ 2018)	MRI at 32 weeks GA					+		Y	1
	MRI at 34 weeks GA					+			
	MRI at 36 weeks GA	+				+			
	MRI at 38 weeks GA					+			
	MRI at day 1	+							
	MRI at day 17	+							
	MRI at day 27	+							
	MRI at 24 months								
5 (Gümüş et al.^18^ 2010)	US at 35 weeks GA							Y	1
	MRI at day 3	+		+	+	+			
6 (Carmi-Nawi et al.^19^ 2011)	US at 35 weeks GA			+	+			Y	1
	CT at day 7			+	+				
	MRI at 5 months				+				
7 (Higuchi et al.^20^ 2014)	US at day 1			+				Y	2
	MRI at 4 hours	+	+	+					
	MRI at day 3.7	+	+	+					
8 (Higuchi et al.^20^ 2014)	US and MRI at day 1	+		+				Y	1
	MRI at day 24	+		+	+				
9 (In-house C2)	US at 12 weeks GA								1
	US at 20 weeks GA								
	US at 22 weeks GA								
	US at 12 hours	+							
	MRI at day 3	+	+						
10 (In-house C3)	US at 20 weeks GA								**1**
	US at day 2	+							
	MRI at day 5	+	+						
11 (Kikuchi et al.^21^ 2012)	MRI and CT at day 3	+	+						1
	MRI at 1 month			+	+				
	MRI at 1 year			+	+				
12 (Bayram et al.^22^ 2013)	US at day 5	+							1
	MRI at day 27			+	+				
13 (Sie et al.^23^ 2010)	US at day 1								1
	US at day 2	+							
	MRI at day 3	+	+						
14 (Per et al.^24^ 2007)	MRI at day 17			+	+		s.h.	Y	1
15 (Veldman et al.^25^ 2010)	MRI at day 6	+							1
	US at 1 month	+			+				
16 (In-house C4)	US day 3	+	+		+			Y	2
	MRI day 6	+	+	+	+				
17 (Serrano et al.^26^ 2007)	US at day 3	+							2
	US at day 5								
	US at day 8	+							
	MRI at day 12	+	+						
	US at day 15	+		+	+				
18 (Vijayakumar et al.^27^ 2011)	MRI at day 7	+	+		+			Y	2
	MRI at 3 months			+	+				
19 (Lin et al.^28^ 2021)	MRI at day 3	+							1
	MRI at day 23	+		+					
20 (Nagappa et al.^29^ 2015)	MRI at 4 months				+				1
21 (Yoganathan et al.^8^ 2018)	MRI at 2 weeks		+	+	+				2
22 (Yoshimura et al.^30^ 2019)	MRI at 12 months	+			+				1
	MRI at 12 years				+				
23 (Yoshimura et al.^30^ 2019)	MRI at 4 months				+	+			1
24 (Stence et al.^31^ 2013)	MRI at day 27				+				1
	MRI at 5 months				+	+	s.h.		
25 (Lee et al.^32^ 2021)	MRI at 5 months		+					N	3
	MRI at 4 years		+		+	+			
	MRI at 7.6 years		+			+			
26 (Sass et al.^33^ 2010)	MRI at day 40	+	+						**1**
	MRI at 2 months				+	+			
27 (Vijayakumar et al.^27^ 2011)	MRI at 10 months					+			1
	MRI at 26 months					+			
	MRI at 33 months					+			
	MRI at 51 months					+			
28 (Lee et al.^32^ 2021)	MRI at 19 months		+					N	2
	MRI at 25 months		+						
29 (Vijayakumar et al.^27^ 2011)	MRI at 5.5 years	+				+			1
30 (Alkufri et al.^34^ 2013)	MRI at 6 years				+				3
	MRI at 23 years	+		+					
	MRI at 23.5 years	+	+						

*GA*, gestational age; *s.h.*, subdural hematoma.

a Fossa abnormalities were not assessed in determining the number of events.

### Minimal number of events

Based on the aggregated imaging data, 5 of 21 (24%) (cases 7, 16-18 and 21) of classical cases had multiple events (Table 2, Typical cerebral MoCD damage detected by imaging modalities). In an additional case (case 9), multiple events were apparent based on the postmortem evidence. When presuming that onset of symptoms signified a recent event, 11 of 21 classical cases (52%) (cases 2, 3, 5-9, 16-18, and 21) had multiple events. Three of 9 (33%) late-onset cases also exhibited multiple events. See Supplemental File 2 for full explanations of the deduced number of events per case.

## Discussion

The clinical symptoms that arise in the first days of life in patients with MoCD, suggest a postnatal disease onset, neatly aligning with the notion of toxic effects on the brain of postnatal sulfite accumulation when placental clearance ceases. This meta-analysis of the radiological data of patients with MoCD challenges this paradigm and indicates that a prenatal onset of brain damage may be the rule rather than the exception. Additionally, it provides evidence that the damage observed shortly after birth can only be explained when presuming more than 1 event in at least 27% of cases. Together, these observations indicate that birth is just one of life’s challenges that can provoke cerebral damage in MoCD. This novel paradigm aligns with the observations in affected children, usually late-onset cases, surviving after birth in which repeated clinical events have been described.^8^^,^^18^^,^^19^^,^^22^^,^^24^^,^^27^^,^^29^^,^^31^^,^^32^^,^^34^^,^^35^

The observation of cerebral damage that has already developed before birth in many classical cases should be taken into account when exploring preventive avenues to improve outcome. This is particularly relevant when considering the use of fosdenopterin, which aims to replenish the shortage of cyclic pyranopterion monophosphate, which arises due to MoCD type A. It has been established that fosdenopterin treatment needs to be initiated shortly after the development of symptoms to be efficacious. Although newborn screening is thus unlikely to be of benefit, in utero therapy with cyclic pyranopterion monophosphate, similar to in utero enzyme replacement therapy for infantile-onset Pompe’s Disease, could potentially prevent cerebral damage.^36^^,^^37^

Up to 3 separate events, resulting in cerebral damage, were needed to explain the radiological findings. Multiple events would clarify the discrepancy between a prenatal onset of brain damage and a symptom-free interval of hours to days after birth. It is tempting to speculate that intrauterine symptoms associated with prenatal cerebral damage have faded at the time of birth. It is presently uncertain whether intrauterine symptoms have ceased to exist or never commenced in the first place. We suspect the latter as, in retrospect, mothers of children with MoCD often describe the frequent sensation of fetal hiccups during their pregnancy, which could have been fetal convulsions. The notion that prenatal damage itself no longer causes symptoms at the time of birth is supported by case 1, in whom no overt symptoms were noted after birth despite well documented prenatal onset of brain damage, including cysts and atrophy being observed at 34 weeks of gestation. Along these lines, we suspect that postnatal symptomatology could be, in fact, induced by birth, be it as a first or a second event. The mechanism of these multiple hits and why certain patients are more prone than others is still to be delineated.

This study was subjected to a number of limitations. First, to allow our analyses, we extrapolated insights obtained through serial analyses of HIE, assuming that—given the clinical and radiological similarities between HIE and MoCD—the developmental time for various types of cerebral damage in patients with MoCD to occur would be the same. To establish if the presented deduction tool is accurate, these serial imagings will have to be conducted in future. Second, deducing the likely timing of onset based on radiological data was possible in most but not all cases with MoCD because of the retrospective design of our study. Particularly, in the case of a later debut of the disease with lack of early imaging data and follow-up imaging, made it impossible to state whether pathogenesis could be of an earlier onset. Third, it is currently unknown if there is a difference between the prenatal and postnatal evolution of cerebral damage. Not all types of cerebral damage are distinguishable prenatally (i.e., migrational abnormalities, such as polymirogyria below 30 weeks of gestation and the presence of cysts in white matter is dynamic and may disappear over time). However, the presence of ventriculomegaly, cerebellar hypoplasia, and cysts in the white matter all clearly indicate that injury occurred in utero. Therefore, the specific type of cerebral damage detected by imaging modalities gives a clear indication of the time of onset of the damage.

Aside from imaging abnormalities, typical phenotypic features in MoCD, such as facial dysmorphism, are often described. In our cohort, 9 cases reported MoCD-specific dysmorphic facial features, and an additional 5 cases suffered from lens luxation, usually years after birth. The dysmorphic features might also suggests an antenatal onset of the damaging effects of the disease as sagging cheeks and lens dislocation can both be explained by the disruption of sulfite bridges between collagen strings, decreasing their strength.^38^^,^^39^ The craniofacial dysmorphic features (including enophthalmos, prominent cheeks, coarse facies, and bitemporal narrowing) and microcephaly and cerebellar hypoplasia described in MoCD observed shortly after birth also suggest that fetal development is affected by the disease in utero.^1^ Dysmorphic features and cerebellar abnormalities align with the notion that patients with MoCD have a lifelong risk of damage from early gestation onward.

In conclusion, our data indicate that antenatal damage may be the rule rather than the exception in classical MoCD. The typical cerebral damage that is associated with MoCD seems best to be explained by multiple episodes of metabolic derailment rather than a single one.

## Data Availability

The original contributions presented in the study are included in the supplemental material, and further inquiries can be directed to the corresponding author.

## Conflict of Interest

The authors declare no conflicts of interest.

## Supplementary Material

Supplementary File S2

In-house cases variantvalidator.org
