# Supplementary material for: Timing of cerebral damage in molybdenum cofactor deficiency: A meta-analysis of case reports
Source: Genet Med Open. 2024 May 24;2:101853. doi: 10.1016/j.gimo.2024.101853 (PMC11613691; doi:10.1016/j.gimo.2024.101853)
Supplement: Supplementary File S1 [file mmc1.pdf]

SUPPLEMENTARY FILE – IN-HOUSE CASES 1, 9, 10 and 16

Case 1 was the third child of healthy unrelated Dutch parents. The second child (sister) passed away 5 days postnatally which was at the time attributed to hypoxic ischemic encephalopathy related to placental floor infarction. Fetal growth was monitored by ultrasound due to the recurrence risk of a placental floor infarction. At the age of 34 weeks, ultrasound examination revealed enlarged ventricles, a large cisterna magna with a small cerebellum and evidence of polymicrogyria. These findings, combined with the family history, raised the suspicion of MoCD. Results of amniotic fluid analyses were confirmatory; showing high S-Sulfocysteine 42 µmol/l (ref. non detectable), low cysteine 15 µmol/l (ref. 38-91) and raised taurine 170 µmol/l (ref. 70-142). Additional investigation of cultured amniocytes revealed absence of sulphite oxidase activity. Delivery was induced at 36 4/7 weeks of gestation. Apgar scores were 9 and 10 at 1 and 5 minutes, respectively. Birthweight was 3670 gram (>P97). Physical examination showed full cheeks and deep-set eyes. She passed away at the age of 3 ½ months. Genetic analysis revealed a homozygous pathogenic variant (NM_001358530.2:c.418+1G>A) in *MOCS1* in both case 1 and her deceased sister.

Case 9, a boy and first child of unrelated Dutch parents, was born at 38 + 5/7 weeks of gestation with Apgar scores of 9/10 after respectively 5 and 10 minutes, and birthweight 4032 gram (P97). During the last 3 weeks of pregnancy, mother had reported reduced fetal movements. As of 1 ½ hours postnatally, he exhibited apneas, cyanosis and jitteriness confirmed to be convulsions on a continuous EEG. Ultrasound on day 1 revealed increased echogenicity of both thalami and white matter abnormalities. MRI on day 3 showed increased signal intensity of the white matter, diffusion weighted imaging was profoundly abnormal, indicative of cerebral ischemia. Postnatal metabolic screening revealed grossly elevated xanthine (708, ref. <20.4 mmol/mol creatinine), low uric acid, elevated thiosulfate (20, ref.<2) and S-sulfocysteïne, suggestive of MoCD. Enzymatic analysis revealed a deficiency of sulphite oxidase. He passed away at the age of four days. Post-mortem genetic analysis revealed pathogenic compound heterozygous variants in *MOCS1* (NM_001358530.2:c.418+1G>A and NM_001358530.2:c.956G>A(p.Arg319Gln)).

Case 10, a girl and second child of non-consanguineous Dutch parents, was born at a gestational age of 41 2/7 weeks with Apgar scores of 9 and 10 at 1 and 5 minutes, respectively, and a birthweight of 3620 gram (P50), a length of 53 cm (P50-97) and a head circumference of 34 cm (P10-50). Pregnancy and birth were uneventful, the mother had experienced two miscarriages prior to this pregnancy. Routine ultrasound at 20 weeks was, other than a choroid plexus cyst, unremarkable. The girl did not drink from birth onwards and was admitted within 24 hours because of inconsolable, high-pitched, crying and poor a greyish-yellow skin colour raising the suspicion of sepsis/meningitis for which antibiotics were started. On examination, hypertonia was striking. A few hours later she exhibited seizures. Anticonvulsive therapy was started and she was transferred to a neonatal intensive care unit. Cranial ultrasound revealed generalized edema, with diffuse white matter abnormalities and multiple germinal layer cysts. MRI revealed massive abnormalities of diffusion weighted imaging of the cerebrum, sparing the cerebellum. Specifically, cystic degeneration was seen in the basal ganglia in the left hemisphere with atrophy of the left hemisphere as well. Metabolic screening revealed grossly elevated levels of xanthine (1425, ref. <20.4 mmol/mol creatinine) combined with a low uric acid 246, ref. >381), together suggestive of MoCD. Elevated lactate and glutamate were evident on magnetic spectroscopy. Epilepsy was progressive and refractory to treatment. She passed away 7 days after birth. Post-mortem genetic analysis pathogenic compound heterozygous variants in *MOCS1* (NM_001358530.2:c.418+1G>A and NM_001358530.2:c.377G>A(p.Gly126Asp))) by Sanger sequencing.

Case 16, a girl and the second child of non-consanguineous Dutch parents, was born after an uneventful pregnancy at 39 weeks and 2 days, with a birthweight of 3395 grams (P50). No dysmorphic features were observed, except for a somewhat small fontanel. A few hours after birth, the parents noticed that she was irritable, less alert and exhibited feeding difficulties. A day later she was admitted with a rise in temperature (38° Celsius) and inconsolable crying. On examination, she was agitated and moved vigorously. The patient exhibited drops in saturation (65%). Amplitude integrated EEG showed convulsions from day 2 of life and phenobarbital treatment was initiated (20

mg/kg/day). A cerebral ultrasound was conducted at day 3 of life and showed asymmetrical ventricles (left ventricle was more pronounced than the right), right sided edema with a slight midline shift and lesions in the basal ganglia and periventricular. An MRI performed on day 6 showed areas of restricted diffusion as well as cerebellar hypoplasia, together indicative of MoCD (figure 3). Results of metabolic workup, with a low uric acid (81, ref. >751) and increased levels in xanthine(858, ref. <47), hypoxantine, s-sulfocysteine (38, ref. <5) and alpha-aminoadipine semialdehyde (AASA; 39,7, ref. <1,8) supported this suspicion. Genetic analysis by sanger sequencing later confirmed two pathogenic variants in *MOCS2*, (NM_001358530.2:c.217C>T(p.Arg73Trp) and NM_001358530.2:c.418+1G>A). Seizure activity proved therapy resistant. The patient passed away 9 days after birth.
