# Supplementary material for: Timing of cerebral damage in molybdenum cofactor deficiency: A meta-analysis of case reports
Source: Genet Med Open. 2024 May 24;2:101853. doi: 10.1016/j.gimo.2024.101853 (PMC11613691; doi:10.1016/j.gimo.2024.101853)
Supplement: Supplementary File S2 [file mmc2.pdf]

Case[Ref.]	Cerebral damage could be explained by # number of events	Definite timing of onset of damage explained	
		Timing of onset	Mode of determination onset
1[C1]	1	Prenatal	Cerebral damage present prenatally
2[1]	1	Prenatal	Cerebral damage present prenatally
3[2]	1	Prenatal	Cerebral damage present prenatally
4[1]	1	Prenatal	Cerebral damage present prenatally
5[3]	1	Prenatal	Deduction: cysts and atrophy at day 3
6[4]	1	Prenatal	Cerebral damage present prenatally
7[5]	2; DWI (temporary detectable cerebral damage) at both 4 hours (event 1) and at 3.7 days of age (event 2)	Prenatal	Deduction: cyst on day 1
8[5]	1	Prenatal	Deduction: cyst on day 1
9[C2]	1	Unknown	PS (long-term damage) at 12 hours deduces to a prenatal or perinatal onset
10[C3]	1	Unknown	PS (long-term damage) at day 2 deduces to a prenatal or perinatal onset
11[6]	1	Unknown	PS (long-term damage) and DWI at day 3 deduces to a prenatal or perinatal onset
12[7]	1	Unknown	PS (long-term damage) deduces to a prenatal or perinatal onset
13[8]	1	Unknown	Deduction: PS (long-term damage) at day 2 deduces to a perinatal or postnatal onset.
14[9]	1	Prenatal	Deduction: atrophy on day 17
15[10]	1	Unknown	PS (long-term damage) on day 6 deduces to a

			prenatal or perinatal onset
16[C4]	2; cUS at 3 days showing PS/atrophy and MRI at 6 days of age showing cyst/atrophy (event 1) and acute injury (DWI) (event 2)	Prenatal	Deduction: atrophy on day 3
17[11]	2; PS at day 3 (event 1) and DWI (temporary detectable cerebral damage) at 12 days of age (event 2)	Unknown	PS (long-term damage) on day 3 deduces to a prenatal or perinatal onset
18[12]	2; PS, DWI and atrophy at day 7 of life (event 1) and cysts at 3 months of life (event 2)	Prenatal	Deduction: PS and atrophy on day 7
19[13]	1	Unknown	PS (long-term damage) on day 6 deduces to a prenatal or perinatal onset
20[14]	1	Unknown	Atrophy (long-term cerebral damage) at 4 months deduces to a prenatal, perinatal or postnatal onset
21[15]	2 combination of DWI (Acute detectable cerebral damage) and atrophy (long-term cerebral damage), suggests two separate events	Unknown	Cysts and atrophy (long-term damage) at 2 weeks deduces to a perinatal onsets
22[16]	1	Unknown	Atrophy (long-term damage) at 12 months deduces to a prenatal, perinatal or postnatal onset
23[16]	1	Unknown	PS and atrophy (long-term damage) at 12 months deduces to a prenatal, perinatal or postnatal onset
24[17]	1	Unknown	Atrophy (long-term cerebral damage) at 27 days deduces to a prenatal, perinatal or postnatal onset
25[18]	3; DWI (temporary detectable cerebral damage) occurs at 5 months, 4 years and 7.6 years of age	Postnatal	Cerebral damage (DWI, acute damage) only presented

			postnatally at 5 months of age
26[19]	1	Unknown	Postnatal onset could possibly be compatible with symptoms on day 40
27[12]	1	Unknown	Atrophy (long-term cerebral damage) at 10 months deduces to a prenatal, perinatal or postnatal onset
28[18]	2; DWI (temporary detectable cerebral damage) at both 19 months and 25 months of age	Postnatal	Cerebral damage only postnatal (DWI, acute damage) first at 19 months and no other cerebral damage
29[12]	1	Unknown	PS (long-term cerebral damage) at 5.5 years deduces to a prenatal, perinatal or postnatal onset
30[20]	3; Atrophy (long-term cerebral damage) at 6 years (event 1), PS and cysts (atrophy cannot develop into cysts, thus newly formed damage) at 23 years (event 2) and DWI (temporary detectable cerebral damage) at 23.5 years of age (event 3)	Unknown	Atrophy (long-term cerebral damage) at 6 years deduces to a prenatal, perinatal or postnatal onset

C1-C4: in-house cases

References

1. Lubout CMA, Derks TGJ, Meiners L, Erwich JJ, Bergman KA, Lunsing RJ, et al. Molybdenum cofactor deficiency type A: Prenatal monitoring using MRI. Eur J Paediatr Neurol. 2018;22(3):536-40.
2. Hannah-Shmouni F, MacNeil L, Potter M, Jobling R, Yoon G, Laughlin S, et al. Severe cystic degeneration and intractable seizures in a newborn with molybdenum cofactor deficiency type B. Mol Genet Metab Rep. 2019;18:11-3.
3. Gumus H, Ghesquiere S, Per H, Kondolot M, Ichida K, Poyrazoglu G, et al. Maternal uniparental isodisomy is responsible for serious molybdenum cofactor deficiency. Dev Med Child Neurol. 2010;52(9):868-72.
4. Carmi-Nawi N, Malinger G, Mandel H, Ichida K, Lerman-Sagie T, Lev D. Prenatal brain disruption in molybdenum cofactor deficiency. J Child Neurol. 2011;26(4):460-4.
5. Higuchi R, Sugimoto T, Tamura A, Kioka N, Tsuno Y, Higa A, et al. Early features in neuroimaging of two siblings with molybdenum cofactor deficiency. Pediatrics. 2014;133(1):e267-71.
6. Kikuchi K, Hamano S, Mochizuki H, Ichida K, Ida H. Molybdenum cofactor deficiency mimics cerebral palsy: differentiating factors for diagnosis. Pediatr Neurol. 2012;47(2):147-9.
7. Bayram E, Topcu Y, Karakaya P, Yis U, Cakmakci H, Ichida K, et al. Molybdenum cofactor deficiency: review of 12 cases (MoCD and review). Eur J Paediatr Neurol. 2013;17(1):1-6.
8. Sie SD, de Jonge RC, Blom HJ, Mulder MF, Reiss J, Vermeulen RJ, et al. Chronological changes of the amplitude-integrated EEG in a neonate with molybdenum cofactor deficiency. J Inherit Metab Dis. 2010;33 Suppl 3:S401-7.
9. Per H, Gumus H, Ichida K, Caglayan O, Kumandas S. Molybdenum cofactor deficiency: clinical features in a Turkish patient. Brain Dev. 2007;29(6):365-8.
10. Veldman A, Santamaria-Araujo JA, Sollazzo S, Pitt J, Gianello R, Yaplito-Lee J, et al. Successful treatment of molybdenum cofactor deficiency type A with cPMP. Pediatrics. 2010;125(5):e1249-54.
11. Serrano M, Lizarraga I, Reiss J, Dias AP, Perez-Duenas B, Vilaseca MA, et al. Cranial ultrasound and chronological changes in molybdenum cofactor deficiency. Pediatr Radiol. 2007;37(10):1043-6.
12. Vijayakumar K, Gunny R, Grunewald S, Carr L, Chong KW, DeVile C, et al. Clinical neuroimaging features and outcome in molybdenum cofactor deficiency. Pediatr Neurol. 2011;45(4):246-52.
13. Lin Y, Liu Y, Chen S, Zhu J, Huang Y, Lin Z, et al. A neonate with molybdenum cofactor deficiency type B. Transl Pediatr. 2021;10(4):1039-44.
14. Nagappa M, Bindu PS, Taly AB, Sinha S, Bharath RD. Child Neurology: Molybdenum cofactor deficiency. Neurology. 2015;85(23):e175-8.
15. Yoganathan S, Sudhakar S, Thomas M, Kumar Dutta A, Danda S, Chandran M. Novel Imaging Finding and Novel Mutation in an Infant with Molybdenum Cofactor Deficiency, a Mimicker of Hypoxic-Ischaemic Encephalopathy. Iran J Child Neurol. 2018;12(2):107-12.
16. Yoshimura A, Kibe T, Hasegawa H, Ichida K, Koshimizu E, Miyatake S, et al. The Persistent Generalized Muscle Contraction in Siblings with Molybdenum Cofactor Deficiency Type A. Neuropediatrics. 2019;50(2):126-9.
17. Stence NV, Coughlin CR, 2nd, Fenton LZ, Thomas JA. Distinctive pattern of restricted diffusion in a neonate with molybdenum cofactor deficiency. Pediatr Radiol. 2013;43(7):882-5.
18. Lee HF, Hsu CC, Chi CS, Tsai CR. Genotype-Phenotype Dissociation in Two Taiwanese Children with Molybdenum Cofactor Deficiency Caused by MOCS2 Mutation. Neuropediatrics. 2021.
19. Sass JO, Gunduz A, Araujo Rodrigues Funayama C, Korkmaz B, Dantas Pinto KG, Tuysuz B, et al. Functional deficiencies of sulfite oxidase: Differential diagnoses in neonates presenting with intractable seizures and cystic encephalomalacia. Brain Dev. 2010;32(7):544-9.
20. Alkufri F, Harrower T, Rahman Y, Hughes E, Mundy H, Knibb JA, et al. Molybdenum cofactor deficiency presenting with a parkinsonism-dystonia syndrome. Mov Disord. 2013;28(3):399-401.
